# Is LysM-Cre a good candidate Cre for knocking out Atg5 gene in mice?

**DOI:** 10.3389/fimmu.2022.964496

**Published:** 2022-08-26

**Authors:** Jun-Hao Wen, Dong-Yi Li, Shan Liang, Ji-Xin Tang

**Affiliations:** Guangdong Provincial Key Laboratory of Autophagy and Major Chronic Non-Communicable Diseases, Institute of Nephrology, Affiliated Hospital of Guangdong Medical University, Zhanjiang, China

**Keywords:** Autophagy, Atg5, Cre/LoxP system, LC3–PE, LysM-Cre

## Introduction

Microtubule-associated protein 1 light chain 3 (MAP1LC3/LC3), one of the mammalian homologs of yeast Atg8, is essential for diverse cellular processes, such as autophagy, phagocytosis, and endocytosis (1–3). During these processes, LC3 needs to be activated by many autophagy-related gene (Atg)-coded proteins to covalent binding (lipidation) with phosphatidylethanolamine (PE) (4). Among these Atg proteins, Atg5 and Atg7 are believed to be indispensable for the ligation of LC3 with PE (5). In autophagy, Radović and colleagues generated Atg5- and Atg7-deficient macrophages through crossing LysM-Cre mice with Atg5- or Atg7-floxed mice, respectively, and reported that Atg5, but not Atg7, is indispensable for LC3–PE conjugation in thioglycolate-elicited macrophages (6). However, considering the position of LysM-Cre and Atg5 in mouse chromosomes and the construction method of LysM-Cre transgenic mice, we believe that it is better to select another macrophage-specific Cre, rather than LysM-Cre, to knock out Atg5 gene to obtain a true and reliable conclusion.

## Cre/loxP-mediated recombination system

The Cre/loxP-mediated recombination system, a powerful conditional gene expression and gene deletion tool, has been widely used to test the functions of specific gene products in mouse development and disease (7). Using this system, we can delete the genes of interest in specific cells, tissues, and even the whole organism so as to generate a variety of conditional knockout mouse strains. Moreover, it is also used to generate cell- or tissue-specific reporter mice for lineage tracing. Although the Cre/loxP system is becoming more and more widely used, especially due to the increasing availability of conditioned mouse mutants, there are many factors that need to be considered when using this system (8)—for example, off-target effects or the toxicity of Cre needs to be considered. Moreover, in order to generate conditional knockout mice efficiently, mating between mice with Cre and floxed genes in the same chromosome should be avoided.

To generate cell- or tissue-specific conditional knockout alleles in mice, it is needed firstly to knock in two loxP sites at distant introns within the gene of interest and then cross this knock-in strain with another mouse strain that expresses the Cre recombinase transgene under the control of a cell- or tissue-specific gene promoter. Recently, the Cre/loxP system has been extensively used to explore the gene function in monocytes/macrophages in mouse models. Compared to other immune cell types such as neutrophils, mast cells, and basophils, monocytes/macrophages are a highly heterogeneous cell type which lack specific markers to mark them. Although considerable efforts have been made over the past decade to establish macrophage-specific Cre mice, existing mouse strains are not perfect in terms of depletion efficiency and macrophage-targeted specificity (9).

## LysM-Cre is an effective tool for knocking out genes in macrophages

Lysozymes are antibacterial enzymes in the natural immune system, which are widely found in plants, animals, bacteria, and fungi, playing an antibacterial role by cleaving peptidoglycans of bacterial cell walls (10). While humans have only one lysozyme gene, the mouse genome encodes two highly homologous lysozyme genes, the lysozyme 1 (Lysz1) or P gene (LysP) and lysozyme 2 (Lysz2) or M gene (LysM), which are both located in chromosome 10 and produced by a recent gene replication (11). LysP is specifically expressed in the Paneth cells of the small intestine, whereas LysM is mainly expressed in myelomonocytic cells such as monocytes, macrophages, and granulocytes (11, 12). Therefore, LysM has been regarded as a specific marker for myeloid cells since its discovery.

In 1999, a myeloid-specific Cre-expressing mouse line, LysM-Cre, was generated by Förster and colleagues through directly inserting engineered Cre cDNA into the endogenous lysozyme 2 locus. The LysM-Cre knock-in/knock-out allele contains a nucleus-localized Cre recombinase, which was inserted into the endogenous ATG-start site within the first exon of Lyz2 in mouse chromosome 10, the endogenous Lyz2 gene function was abolished and Cre expression was placed under the control of endogenous Lyz2 promoter/enhancer elements (13). When crossed with a mouse strain that contained a loxP site-flanked gene sequence of interest, Cre-mediated recombination will lead to the loss of function of the targeted gene in the myeloid cell lineage, such as monocytes, mature macrophages, and granulocytes. Therefore, the LysM-Cre strain is believed to be an effective tool for generating macrophage-specific targeted mutants, though LysM is not a specific marker for macrophages.

## Recombination frequency

Due to the exchange of homologous fragments during meiosis, incomplete linked genes always recombine to form a certain proportion of reassortative gametes. The percentage of the number of recombination gametes to the total number of gametes is called the recombination value or recombination frequency. CentiMorgan (cM) is a unit of measurement of recombination frequency, wherein 1 cM is the distance at which recombination occurs once every 100 times. In other words, if two genes are separated by 1 cM, 1% of their offspring will have different allele frequencies than their parents. In fact, the distance between genes can range from 1 to 50 cM, and the smaller the distance, the closer the genes are to each other on the chromosome. A distance of 1 cM indicates that the distances between genes are closely linked, and their positions on a chromosome are relatively close together. In contrast, the 50-cM distance means that the two genes are not linked and are likely to be located on different chromosomes. In general, the smaller the number, the more accurately the distance between two genes can be determined. When the distance between two genes is between 40 and 50 cM, the precise relationship between two genes cannot be determined. Therefore, we do not know if the two genes are linked.

## Discussion

The mouse Atg5 gene also locates in chromosome 10, and the Atg5 protein can associate with Atg12, functioning as an E1-like activating enzyme in a ubiquitin-like conjugating system, which is essential for autophagic vesicle formation (4). Vujić et al. generated mice with a targeted deletion of Atg5 or Atg7 in myeloid cells by crossing LysM-Cre with Atg5- or Atg7-floxed mice, respectively (6). They isolated macrophages lacking Atg5 or Atg7 from these transgenic mice and analyzed the effects of Atg5 and Atg7 on LC3–PE conjugation through drug stimulation *in vitro*. They found that Atg5, but not Atg7, is indispensable for LC3–PE conjugation in thioglycolate-elicited mouse peritoneal macrophages. We agree with Vujić et al. that mammalian Atg5 and Atg7 are not equally important for LC3–PE conjugation, and it may be possible to cause LC3–PE conjugation without Atg7 in some cases.

**Figure 1:**
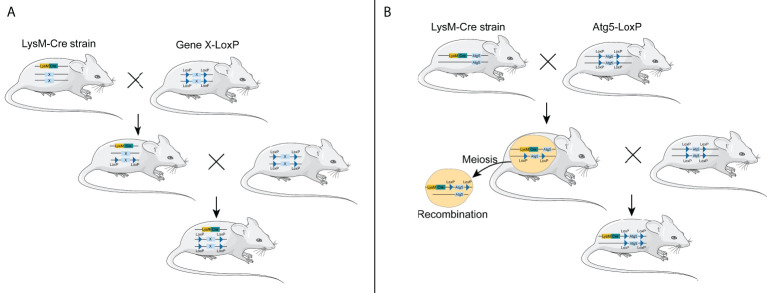
Generation of conditional knockout mice using LysM-Cre mouse strain. **(A)** General process of constructing macrophage-specific conditional knockout mice using LysM-Cre mouse strain through the Cre/loxP system. **(B)** Generation of conditional knockout Atg5 mice using LysM-Cre mouse strain through the Cre/loxP system. LysM-Cre and Atg5 floxed alleles locate in the same chromosome in mice.

However, considering both LysM-Cre- and Atg5-floxed alleles located in chromosome 10 in mice, the LysM-Cre mice are not suitable to cross with Atg5-floxed mice to obtain myeloid Atg5-deficient mice. The Atg5 gene locates in chromosome 10 B2/23.24 cM, while the LysM gene is in chromosome 10 D2/65.34 cM. The distance between the two genes was 95,970 bp (42.1 cM), so whether the two genes are linked remains unknown. Theoretically, it is difficult to obtain myeloid ATG5-deficient mice in this way; however, several research groups have obtained Atg5f/f; LysM-Cre mice by crossing Atg-floxed mice with LysM-Cre mice (14–17), suggesting that the two genes are not linked together and can be recombined during meiosis (**Figure 1**). In addition, Cre insertion results in LysM gene inactivation, so even Atg5-deficient macrophages can be luckily obtained in this way. These macrophages are not just lacking the Atg5 gene; they are also partially lacking the LysM gene. Taking the above-mentioned two points into consideration, we hold that other macrophage-specific Cre lines, instead of LysM-Cre, should be recommended so as to efficiently obtain mice with the Atg5-deficient macrophages.

As we mentioned earlier, there is still no good Cre to knock out genes specifically in macrophages (9, 18) (**Table 1**). Although the LysM-Cre strain is often used to delete genes in macrophages in mouse models, but it can also result in the loss of function of genes in granulocytes, dendritic cells (DCs), and even neurons (13, 19). Colony-stimulating factor 1 receptor (CSF1R), the receptor of colony-stimulating factor 1, is a transmembrane tyrosine kinase expressed at the cell surface of all the mononuclear phagocytes in mice, including monocytes and macrophages. In 2010, Deng et al. generated the Csf1r-Cre mouse strain, which can express optimized Cre recombinase under the direction of the murine Csf1r promoter (20). Csf1r-Cre is believed to be a good tool to efficiently delete genes in macrophages of various mouse tissues, such as the kidney, liver, heart, and so on. However, the specificity of this Cre line is not high and can also delete genes in other cells, such as granulocytes, DCs, and even T lymphocytes (20). The CD11b-Cre mouse strain was created by Ferron et al. in 2005; it can be used to conditionally knock out genes in the hematopoietic myeloid–osteoclast lineage (21), but it was supposed to be an unreliable strain as an inconsistent deletion between littermates was observed (5). F4/80, a cell surface glycoprotein with seven transmembrane regions, is mainly expressed in the tissue macrophages, such as Kupffer cells and peritoneal macrophages (22, 25). By introducing the Cre recombinase cDNA into the first coding exon of F4/80 in chromosome 17, Schaller et al. generated the F4/80-Cre mouse strain in 2002 (22). However, the F4/80-Cre stain knock-out efficiency is not very high; it can only delete genes in a fraction of peritoneal macrophages. Therefore, this strain was not often used in generating a conditional knockout mouse model (9, 18). CX3C chemokine receptor 1, the receptor of chemokine CX3CL1, is primarily expressed in the mononuclear phagocyte system, including monocytes, macrophages, and DCs. In 2013, Yona et al. generated the Cx3cr1-Cre mice by inserting Cre recombinase into the Cx3cr1 loci in chromosome 9 (23). The deletion efficiency of this Cre is high in peritoneal macrophages at about 70–80% but relatively low (40–60%) in splenic macrophages and peripheral blood monocytes. Besides monocytes/macrophages, the Cx3cr1-Cre strain can also delete genes in mast cells and DCs, but not in granulocytes (18, 23). By using the human CD68 promoter, Franke et al. generated a CD68-Cre mouse strain which can delete genes not only in monocytes and macrophages but also in a portion of epithelial cells and keratinocytes (24).

**Table 1:** Currently available macrophage-specific Cre strains.

Cre strain	Mouse name	MGI number	Published specificity	Reference
LysM-Cre	Lyz^2tm1(cre)Ifo^	1934631	Macrophages and granulocytes, dendritic cells, and even in neurons	13, 19
Csf1r-Cre	Tg(Csfr-icre)1Jwp	4429470	Macrophages, granulocytes, DCs, and even in T lymphocytes	20
CD11b-Cre	Tg(ITGAM-cre)2781Gkl	3629092	Hematopoietic myeloid–osteoclast lineage (transgene on autosome)	21
F4/80-Cre	Emr1^tm1(cre)Kpf^	2429642	Tissue macrophages, peritoneal macrophages (knock-out efficiency is low)	22
Cx3cr1-Cre	Cx3cr1tm1.1(cre)Jung	5467983	Tissue macrophages and monocytes, mast cells, and DCs	18, 23
CD68-Cre	Not listed	Not listed	Macrophages, bone marrow, andsome epithelial cell types	24

Cre strain refers to the common name used in studies, and the mouse name means official gene name. Further information about the Cre strains is available *via* accessing the Mouse Genome Informatics database (www.informatics.jax.org) using the MGI number.

In terms of Atg5 gene knockout, we recommend avoiding the use of LysM-Cre, as it is located in the same chromosome as Atg5, but we recommend selecting another macrophage-specific Cre, such as Csf1r-Cre, Cx3cr1-Cre, or CD68-Cre. Considering the limitations of these macrophage-specific Cre mouse strains, it is preferable to select at least two different Cre to knock out the genes in macrophages. When using knock-in/knock-out Cre, such as LysM-Cre and Cx3cr1-Cre, there should be a stricter control group. Besides the f/f control group, there should be a Cre-only control group, and the Cre should be heterozygous.

## Author contributions

J-HW, D-YL, SL, and J-XT wrote the first draft of the manuscript. All authors contributed to the article and approved the submitted version.

## Funding

This work was supported by the Natural Science Foundation of Guangdong Province (2019A1515110152) and the Discipline Construction Project of Guangdong Medical University (4SG21229G).

## Conflict of interest

The authors declare that the research was conducted in the absence of any commercial or financial relationships that could be construed as a potential conflict of interest.

## Publisher’s note

All claims expressed in this article are solely those of the authors and do not necessarily represent those of their affiliated organizations, or those of the publisher, the editors and the reviewers. Any product that may be evaluated in this article, or claim that may be made by its manufacturer, is not guaranteed or endorsed by the publisher.
